# Follicular Thyroid Carcinoma Metastatic to the Kidney: Report of a Case with Cytohistologic Correlation

**DOI:** 10.1155/2015/701413

**Published:** 2015-10-13

**Authors:** Vikas Nath, Mithra Baliga, Jack Lewin, Frederico Souza, Israh Akhtar

**Affiliations:** ^1^Department of Pathology, University of Mississippi Medical Center, 2500 North State Street, Jackson, MS 39216, USA; ^2^Department of Radiology, University of Mississippi Medical Center, 2500 North State Street, Jackson, MS 39216, USA

## Abstract

Here we report a case of a 45-year-old female who underwent thyroidectomy for thyroid cancer and presented 20 years later with a left renal mass. CT-guided core biopsy was performed, and imprints and histologic sections of the biopsy showed cells resembling thyroid follicular cells with a background containing colloid. Immunohistochemistry revealed positivity for thyroglobulin and thyroid transcription factor 1, consistent with metastatic follicular thyroid carcinoma (FTC). The patient later underwent radical nephrectomy; histologic sections of the resected tumor revealed an encapsulated lesion morphologically similar to the biopsy specimen. Thyroid metastases to the kidney are extremely rare and are often detected during postthyroidectomy surveillance by elevation in thyroid hormone levels, ^131^I scintigraphy, or ^18^F-fluorodeoxyglucose uptake in positron emission tomography studies. Treatment involves total thyroidectomy, resection of the metastatic foci, and ^131^I therapy. The differential diagnoses of renal metastasis of FTC include the encapsulated follicular variant of papillary thyroid carcinoma (PTC), which possesses some of the nuclear features seen in conventional PTC but may occasionally be indistinguishable from FTC in cytologic preparations, and renal lesions such as benign thyroidization of the kidney and thyroid-like follicular carcinoma of the kidney, which mimic FTC in histologic appearance but do not stain with thyroid markers.

## 1. Introduction

Follicular thyroid carcinoma (FTC) is the second most common type of thyroid cancer, after papillary thyroid carcinoma (PTC), accounting for approximately 10% of all malignant thyroid tumors. It has a predilection for women and is more common in areas of endemic goiter [1, 2]. FTC is defined as a malignant epithelial tumor with follicular differentiation that (1) lacks the nuclear features associated with PTC and (2) demonstrates capsular and/or vascular invasion, extrathyroidal extension, or lymphatic or distant metastases [1, 3–6]. FTC is considered a more aggressive tumor than PTC as it often presents at a higher stage, with distant metastases in 25 to 30% of cases [2, 7]. Unlike PTC, which tends to metastasize to cervical lymph nodes, FTC more commonly presents with hematogenous metastases to lung and bone [2, 7, 8]; however, other metastatic sites have been reported as well. We report a case of FTC metastatic to the kidney in a patient presenting with a left renal mass, which was diagnosed by touch imprint cytology and histologic sections of a CT-guided renal core biopsy.

## 2. Case Report

**Figure 1:**
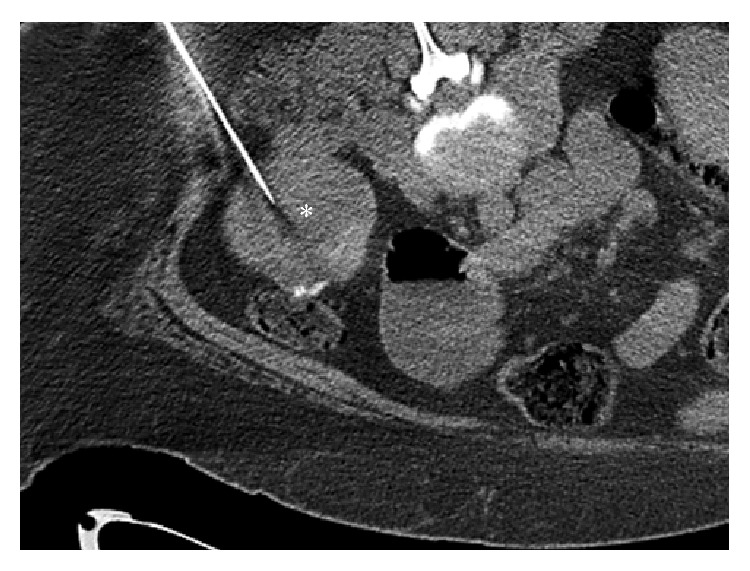
Axial noncontrast CT of the abdomen and pelvis demonstrated a 7.5 × 6.8 cm homogeneous, predominantly exophytic mass arising from the inferior pole of the left kidney. CT-guided biopsy of the left renal mass was performed with a large needle (18 G × 15 cm) (*asterisk*) with the patient in a prone position.

A 45-year-old African American female presented for evaluation of a left renal mass. She did not complain of any urinary symptoms such as flank pain or hematuria, and her past medical history was significant for type 2 diabetes mellitus, gastric bypass surgery, and thyroid cancer diagnosed at an outside hospital 20 years previously, which was treated by total thyroidectomy. However, the histologic type of the thyroid tumor was unknown, and neither the outside medical records nor histologic sections of the thyroid tumor were available for review. The patient's primary care physician had told her that imaging studies of her left kidney were suspicious for renal cell carcinoma and had suggested that she undergo nephrectomy. A CT of the abdomen and pelvis revealed a 7.5 × 6.8 cm, exophytic, homogeneously enhancing mass in the inferior pole of the left kidney. There was no evidence of hydronephrosis or nephrolithiasis, and the urinary bladder, ureters, and adrenal glands were all unremarkable. A percutaneous CT-guided core biopsy of the mass was performed (Figure 1), and imprints and histologic sections of the biopsy specimen were evaluated and deemed positive for metastatic FTC.

Nine months following biopsy, the patient underwent left radical nephrectomy, and examination of histologic sections of the resection specimen confirmed the cytologic diagnosis made on the core biopsy specimen. She did well in the immediate postoperative period, with no evidence of wound infection, and was discharged. Three years later, she was admitted for malnutrition and dehydration due to an anastomotic leak from her previous gastric bypass surgery. She was discharged after a 2-month inpatient stay, during which she underwent gastrostomy tube placement, and has not been hospitalized since. A repeat CT at that time showed several hypodense lesions in the right kidney, as well as one of intermediate density measuring up to 1 cm in greatest dimension that had remained stable since the previous CT, 3 years earlier. The patient was subsequently lost to follow-up.

## 3. Cytohistologic Findings

**Figure 2:**
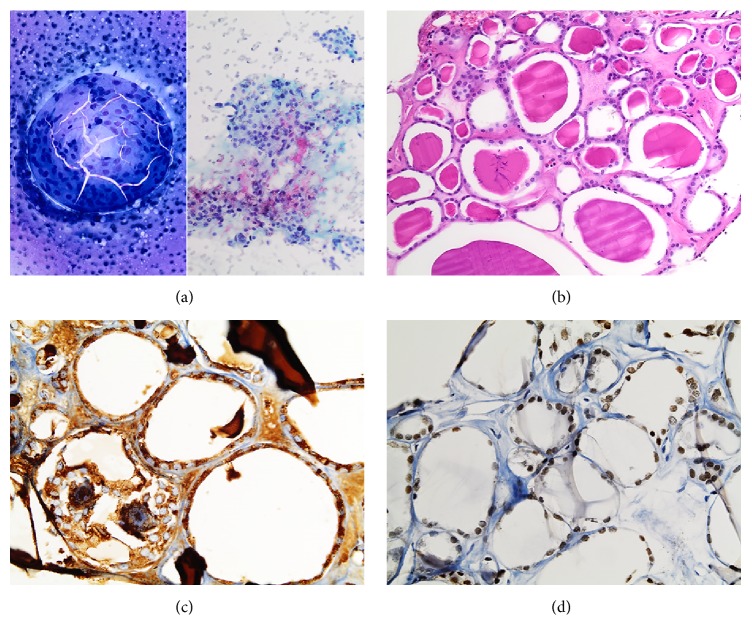
Core biopsy imprints of the left renal mass showed an abundance of cells that resembled thyroid follicular cells arranged in large sheets (a) in a background containing proteinaceous material (Diff-Quik, 200x) (*left*). The cells possessed round to oval nuclei, uniformly distributed chromatin, and inconspicuous nucleoli, without any nuclear features of PTC such as longitudinal grooves, nuclear clearing, or intranuclear pseudoinclusions (Papanicolaou, 200x) (*right*). Histologic sections of the biopsy specimen showed variably sized colloid-filled follicles lined by flattened follicular cells, resembling normal thyroid (H&E, 200x) (b). The thyroid origin of the tumor was confirmed by immunohistochemical stains that demonstrated positive cytoplasmic expression of thyroglobulin (400x) (c) and positive nuclear expression of TTF-1 (400x) (d).

A CT-guided core biopsy of the renal mass was performed, and immediate assessment was done using Diff-Quik stain. Touch imprints of the mass showed numerous cells that were arranged in large sheets and cohesive clusters (Figure 2(a)), with proteinaceous material in the background; these features were highly suggestive of a thyroid origin. However, at the time when on-site evaluation of the imprints was performed, the patient's history of thyroid cancer was not known; therefore, a benign kidney lesion was the favored interpretation initially. Papanicolaou stain revealed sheets of monomorphic cells with round to oval nuclei, uniformly distributed chromatin, and inconspicuous nucleoli. Histologic sections of the biopsy specimen showed variably sized follicles lined by an attenuated layer of thyroid follicular cells and filled with colloid, closely resembling normal thyroid gland (Figure 2(b)). Immunohistochemistry (IHC) revealed positive cytoplasmic staining for thyroglobulin (Figure 2(c)) and positive nuclear staining for thyroid transcription factor 1 (TTF-1; Figure 2(d)) in the nuclei of the follicular cells, thus confirming a thyroid origin. Touch imprint cytology and histologic features showed that the cells had morphology compatible with FTC such as a repetitive microfollicular pattern, round nuclei with irregular chromatin, uniform nuclear membranes, and absent nucleoli. Features that were characteristic of PTC such as longitudinal grooves, nuclear membrane irregularities, nuclear clearing, or intranuclear pseudoinclusions were not seen. Due to these findings, a diagnosis of metastatic FTC was rendered. In the course of further workup of the patient, it was discovered that she had a history of thyroid cancer that was treated with total thyroidectomy at another institution; however, medical records and slides from the outside hospital were not available for review, and so the histology of her primary tumor was uncertain.

**Figure 3:**
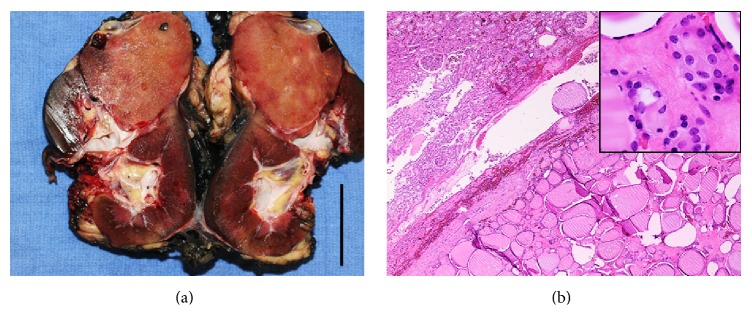
The left radical nephrectomy specimen was bisected to reveal a pink-tan, well-circumscribed mass in the inferior pole (*bar*, 5 cm) (a). Histologic sections of the resection specimen showed follicles filled with colloid and lined by thyroid follicular cells, separated from the adjacent renal parenchyma by a fibrous capsule (H&E, 40x) (b). Higher magnification revealed monomorphic cells with bland nuclei, similar to those in the biopsy specimen, with none of the nuclear features associated with PTC (H&E, 400x) (*inset*).

The surgical resection specimen consisted of a left radical nephrectomy that was bisected to reveal a large, well-circumscribed mass in the inferior pole with spongy pink-tan cut surfaces and areas of hemorrhage and cystic degeneration (Figure 3(a)). The mass abutted the renal capsule but did not invade into the perinephric fat; neither was there tumor involvement of the renal pelvis, renal vein, or adrenal gland. The uninvolved renal parenchyma was unremarkable. Examination of histologic sections of the tumor showed a mass composed of colloid-filled thyroid follicles lined by flattened cells with scant cytoplasm and small round nuclei, which was separated from the adjacent renal parenchyma by a fibrous capsule (Figure 3(b)). There was no evidence of lymphovascular invasion, and the surgical margin, renal vascular margin, and ureteral margin were all negative for tumor. IHC for thyroglobulin and TTF-1 showed the same pattern of expression in the malignant follicular cells as in the core biopsy specimen, thereby providing histologic confirmation of the cytologic diagnosis of metastatic FTC.

## 4. Discussion

Renal metastases of well-differentiated thyroid carcinoma are exceedingly rare, with fewer than 20 cases reported in the English-language literature [1, 8–11] and another 30 in the Japanese literature [1, 8]. By definition, FTC is a histologic diagnosis that requires demonstration of capsular and/or vascular invasion. This essentially precludes diagnosis of a thyroid nodule as FTC by fine-needle aspiration cytology [3, 4], which is why the Bethesda System for Reporting Thyroid Cytopathology allows a cytologic specimen of FTC to be categorized, at most, as a “follicular neoplasm/suspicious for a follicular neoplasm” [12]. Nevertheless, there are certain cytologic features that favor FTC over its benign counterpart, follicular adenoma (FA). For example, aspirate smears of FA have been shown to demonstrate cells in a follicular pattern and to contain colloid; indeed, the presence of a macrofollicular pattern coupled with abundant, watery colloid is a strong indicator that a thyroid nodule is benign. By contrast, FTC may demonstrate a repetitive microfollicular pattern or some other cytologic architecture such as a solid, trabecular, or cribriform pattern; in addition, the presence of nuclear atypia, high nuclear/cytoplasmic ratio, and coarse chromatin suggests malignancy. If cytologic assessment of malignancy is to be performed on a follicular neoplasm, it is better done on poorly differentiated tumors, which possess more of the above-mentioned features, than on well-differentiated FTC, which may mimic FA or even normal thyroid [3].

A second, and more diagnostically challenging, lesion in the differential diagnosis of metastatic FTC is PTC, specifically the encapsulated follicular variant of PTC (FVPTC). This variant resembles FTC not only in the sense of being well circumscribed with a fibrous capsule, but also in its behavior of metastasizing to lung and bone by the hematogenous route, rather than the lymphatic route as is the case with conventional PTC [6]. In addition, it shares several genetic abnormalities with FTC, including* RAS* mutations and* PAX8/PPARγ* rearrangements [6, 13]. Diagnosis of FVPTC by fine-needle aspiration cytology is difficult, as this tumor possesses some, but not all, of the nuclear features associated with PTC. Classically, cytologic preparations of conventional PTC are characterized by papillary architecture with central fibrovascular cores; the accompanying nuclear features include nuclear enlargement, nuclear overlap, chromatin clearing (“Orphan Annie nuclei”), longitudinal grooves, and intranuclear pseudoinclusions [3]. In FVPTC, the most common features are nuclear overlap and chromatin clearing, which are seen in 80% of cases. By contrast, nuclear grooves are present in only 12% of cases and pseudoinclusions in only 5%. In some cases, cells with atypical nuclei are completely absent in cytologic preparations, and the diagnosis of FVPTC can only be made on the basis of histology; the most common cytologic pattern in these specimens is the microfollicular one [13], which, as previously mentioned, is also frequently present in aspirate smears of FTC. FVPTC cytologic specimens are frequently classified under either the “follicular neoplasm/suspicious for a follicular neoplasm” or “suspicious for PTC” category within the Bethesda System, depending on the degree of atypia present [12].

One method that may be used to evaluate diagnostically difficult specimens is IHC, as the triad of galectin-3, Hector Battifora mesothelial cell 1 (HBME-1), and cytokeratin 19 (CK19) is well established as a means of differentiating between various thyroid neoplasms [14, 15]. Galectin-3, a cytoplasmic stain, has high sensitivity and specificity for malignant tumors, particularly PTC and FVPTC, though it is frequently expressed in FTC as well; FA, however, is negative. HBME-1, a nuclear stain, likewise is highly sensitive and specific for thyroid carcinoma. CK19, a membranous stain, shows strong, diffuse positivity in PTC—which includes FVPTC [14]—and is either absent or only focally present in FA and FTC; however, exceptions to this rule do exist [14, 15], and for this reason a combination of immunostains is usually preferable to ordering any individual stain alone. In addition to the above three, several other immunostains have been cited as useful in distinguishing benign from malignant thyroid lesions. Among these is the CD44 isoform CD44v6, whose expression correlates with metastatic potential and which has been shown to be negative in follicular adenoma but positive in several thyroid malignancies; membranous and cytoplasmic CD44v6 positivity has been documented in PTC [14]. Fibronectin 1 and CITED-1 (CBP/p300-Interacting Transactivators with glutamic acid [E] and aspartic acid [D]-rich C-terminal domain) are two other markers that are more highly expressed in malignant thyroid lesions—particularly PTC—than in benign ones; the former shows membranous and cytoplasmic positivity and the latter nuclear and cytoplasmic positivity [16]. We did not order IHC other than thyroglobulin and TTF-1 in this case, as we were able to make the diagnosis of metastatic FTC purely on the basis of cytohistologic and clinical features.

In patients with FTC who have not undergone thyroidectomy, the primary thyroid tumor may present as a neck mass [9, 17]. Presenting symptoms of renal metastases vary, depending on whether the metastasis is isolated to the kidney or involves multiple organs. As a general rule, when tumors metastasize to the kidney, the lesions are multifocal and often bilateral. However, several case reports exist of metastatic FTC presenting as a solitary renal mass [1, 9, 10, 18, 19]; these patients may present with flank pain and/or microscopic hematuria [1, 7, 18]. In patients with an established diagnosis of FTC, postthyroidectomy surveillance is performed by measurement of serum thyroglobulin levels, ^131^I scintigraphy, and positron emission tomography (PET) using an ^18^F-fluorodeoxyglucose- (FDG-) labeled tracer for detection of metastatic foci. Case reports exist of renal metastases of FTC detected by a rise in serum thyroglobulin, areas of increased ^131^I uptake, or FDG-avid foci on PET indicating increased metabolic activity [1, 9, 10, 17], in addition to the presence of a mass on conventional or contrast-enhanced CT. It is not unheard of for patients to present with metastases a decade or more after thyroidectomy [7, 18, 19]. Treatment of metastatic FTC consists of total thyroidectomy, regional lymph node dissection, surgical resection of the metastatic foci with clear margins, and ^131^I radioablative therapy for removal of any residual disease or micrometastases [1, 8–10, 19]. Levothyroxine is also given, with the intent of both replacing thyroid hormone and suppressing thyroid-stimulating hormone levels [9, 17]. The prognosis of metastatic well-differentiated thyroid carcinoma is variable, but on average 50 to 75% of patients die within 5 years of diagnosis [9, 19]; however, younger patients may respond better, especially if aggressive surgical excision is combined with ^131^I radioablation [19]. Radical, or at least partial, nephrectomy to reduce tumor burden is highly recommended to manage solitary late metastases of thyroid carcinoma to the kidney [9, 18].

## 5. Conclusion

Given the rarity of thyroid metastases to the kidney, this case was a diagnostic challenge. Her history of thyroid carcinoma, which was not known to us during our initial assessment of her renal core biopsy, later proved invaluable in directing our approach to formulate a diagnosis, along with the help of immunostains. The histologic appearance of the tumor was suggestive of metastatic thyroid carcinoma, which we were able to confirm using IHC for thyroglobulin and TTF-1. The nuclear features of PTC are readily appreciated on cytologic as well as histologic specimens and were not seen in our case; this was helpful in ruling out FVPTC, which was also in the differential diagnosis. Metastatic tumors in the kidney can be difficult to diagnose on cytologic specimens, and accordingly the differential diagnosis must be wide. Cytohistologic examination has certain limitations when it comes to rare lesions; however, an extensive clinical workup and ancillary studies are useful in formulating a diagnosis. Cases like this enrich the cytology literature by providing awareness of thyroid carcinomas metastasizing to the kidney.
